# Expression profiling of DNA methylation-mediated epigenetic gene-silencing factors in breast cancer

**DOI:** 10.1186/1868-7083-6-20

**Published:** 2014-10-13

**Authors:** Swayamsiddha Kar, Dipta Sengupta, Moonmoon Deb, Arunima Shilpi, Sabnam Parbin, Sandip Kumar Rath, Nibedita Pradhan, Madhumita Rakshit, Samir Kumar Patra

**Affiliations:** 1 Epigenetics and Cancer Research Laboratory, Biochemistry and Molecular Biology Group, Department of Life Science, National Institute of Technology, Rourkela, Odisha 769008, India

**Keywords:** Epigenetics, DNA methylation, DNA methyltransferases, Methyl-CpG-binding domain proteins, Gene silencing, Cancer

## Abstract

**Background:**

DNA methylation mediates gene silencing primarily by inducing repressive chromatin architecture via a common theme of interaction involving methyl-CpG binding (MBD) proteins, histone modifying enzymes and chromatin remodelling complexes. Hence, targeted inhibition of MBD protein function is now considered a potential therapeutic alternative for thwarting DNA hypermethylation prompted neoplastic progress. We have analyzed the gene and protein expression level of the principal factors responsible for gene silencing, that is, DNMT and MBD proteins in MCF-7 and MDA-MB-231 breast cancer cell lines after treatment with various epigenetic drugs.

**Results:**

Our study reveals that the epigenetic modulators affect the expression levels at both transcript and protein levels as well as encourage growth arrest and apoptosis in MCF-7 and MDA-MB-231 cells. AZA, TSA, SFN, and SAM inhibit cell growth in MCF-7 and MDA-MB-231 cell lines in a dose-dependent manner, that is, with increasing concentrations of drugs the cell viability gradually decreases. All the epigenetic modulators promote apoptotic cell death, as is evident form increased chromatin condensation which is a distinct characteristic of apoptotic cells. From FACS analysis, it is also clear that these drugs induce G_2_-M arrest and apoptosis in breast cancer cells. Further, transcript and protein level expression of MBDs and DNMTs is also affected - after treatment with epigenetic drugs; the level of transcripts/mRNA of MBDs and DNMTs has consistently increased in general. The increase in level of gene expression is substantiated at the protein level also where treated cells show higher expression of DNMT1, DNMT3A, DNMT3B, and MBD proteins in comparison to untreated cells. In case of tissue samples, the expression of different DNMTs is tissue stage-specific. DNMT1 exhibits significantly higher expression in the metastatic stage, whereas, DNMT3A and DNMT3B have higher expression in the primary stage in comparison to the metastatic samples.

**Conclusion:**

The epigenetic modulators AZA, TSA, SFN, and SAM may provide opportunities for cancer prevention by regulating the components of epigenetic gene-silencing machinery especially DNMTs and MBDs.

## Background

DNA methylation is one of the principal epigenetic enforcers participating in cell-specific regulation of transcriptional activity. DNA methylation basically acts as a gene-silencing mechanism to turn off specific genes and bring about functional re-orientation of the genomic data at crucial junctures during development and differentiation [1,2]. Methylation of DNA is a post-synthetic process catalyzed by a family of dedicated enzymes known as DNA Methyltransferases (DNMTs). DNMT1, DNMT3A, and DNMT3B methylate the C_5_ of cytosine residue specifically at CpG rich promoter sequences in the presence of cofactor S-Adenosyl methionine (SAM) which donates the -CH_3_ group and is converted to S-Adenosyl homocysteine (SAH) [3,4]. DNA methylation plays an important role in main tenance of genome integrity (by silencing of repetitive sequences, endogenous retroviruses, selfish genetic elements like transposons) and contributes significantly towards X-chromosome inactivation, tissue-specific gene expression, and induction of stem cell differentiation [5-8].

Although, it was established very early that DNA methylation is implicated in gene silencing, the exact mechanism via which this epigenetic tag is co-related with transcriptional inactivation remained a topic of intense speculation. It was assumed that methylated cytosines sterically hinder the binding of transcription factors and RNA polymerase II to their cognate recognition sequences. Also, methylation of DNA resulting in nucleosomal repositioning and high-order chromatin remodeling leads to formation of inactive heterochromatin compartments which further obstructs the gene expression circuit [9,10]. However, a strong connection between DNA methylation and subsequent gene silencing was confirmed after the discovery of a family of conserved proteins, the methyl-CpG-binding domain (MBD) proteins [11-14]. The MBD protein family comprises of five prominent nuclear proteins MeCP2, MBD1, MBD2, MBD3, and MBD4 who are tagged as the epigenetic readers of the methyl signature propagated by the DNMTs [15]. MBD proteins interact with the DNA around the methylated cytosine bases to maintain or alter nucleosomal architecture and direct gene-expression programs. In addition to denying access to the regulators of the transcriptional machinery, MBD proteins also recruit histone deacetylases (HDACs) and chromatin remodeling complexes such as NuRD, SWI/SNF, and Mi-2 to methyl-CpG-enriched promoters in the genome to modulate the chromatin structure and repress transcription [16-19].

**Figure 1:**
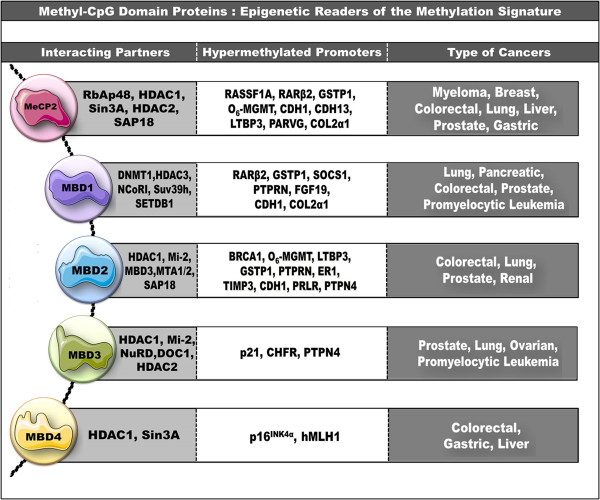
**The figure represents the MBD protein family comprising of five prominent nuclear proteins MeCP2, MBD1, MBD2, MBD3, and MBD4 and the protein partners they interact with such as histone deacetylases (HDACs) and chromatin remodeling complexes such as NuRD, SWI/SNF, and Mi-2.** The figure also depicts the various hypermethylated promoters to which the different MBD proteins bind and mediate gene silencing in various cancer types. While MeCP2, MBD2, MBD3, and MBD4 interact with HDACs to bring about transcriptional repression, MBD1 binds with DNMT1 and acts to silence the hypermethylated promoter regions. Thus, together DNMT and MBD proteins supervise the epigenetic regulatory system to bring about transcriptional incompetence and lead to tumorigenic progression (Adapted from [26,28,29]).

DNA methylation patterns are profoundly altered in malignant cells, characterized by paradoxical gene-specific regional hypermethylation and global hypomethylation of the genome [20,21]. While global genomic demethylation is mainly responsible for oncogene activation and chromosomal instability, DNA methylation arbitrated promoter CpG island hypermethylation leads to silencing and inactivation of tumor suppressor genes, thus paving the way for neoplastic transformation [22-24]. The hypermethylation-induced silencing of tumor suppressor genes in cancer cells is mediated by MBD proteins which bind to the methylated promoters, preventing their transcriptional activation in time to stop cancer [25-31]. MBD protein occupancy of hypermethylated promoters of tumor suppressor genes, cell cycle regulatory, and DNA repair genes followed by their silencing has been reported in a number of cancers (Figure 1). These observations have corroborated the hypothesis that MBD proteins act as the conduit that links DNA hypermethylation and transcriptional misregulation with neoplasia. Therefore, targeted inhibition of MBD proteins is now considered as a means of unmasking silenced genes and initiating transcriptional activity in a bid to counterattack malignant transformation [32].

In view of this, treatment with inhibitors of DNA methylation and histone deacetylation can reactivate epigenetically silenced tumor suppressor genes and successively restore normal gene function such as induction of growth arrest and apoptosis in cancer cells [33]. In this study, we have analyzed the gene and protein expression profile of DNMT and MBD proteins in two breast cancer cell lines after treatment with various epigenetic drugs such as DNMT and HDAC inhibitors- 5-Aza-2'-deoxycytidine (AZA), Trichostatin A (TSA) and Sulforaphane (SFN), respectively, and modulator SAM. The present study is aimed at investigating the molecular effects of these epigenetic manipulators on the expression and activity of the DNA methylation mediated gene-silencing machinery as well as on cell growth. The study will be useful in providing new insights in regulation of DNMT and MBD function and present new avenues for targeting their activity during transcriptional inactivation and gene silencing. This study will also establish DNA methylation and the crucial elements of epigenetic gene-silencing machinery as novel targets for efficient therapeutic interventions in cancer therapy.

## Results

### Epigenetic modulators inhibit cell growth in breast cancer cell lines in a dose-dependent manner

**Figure 2:**
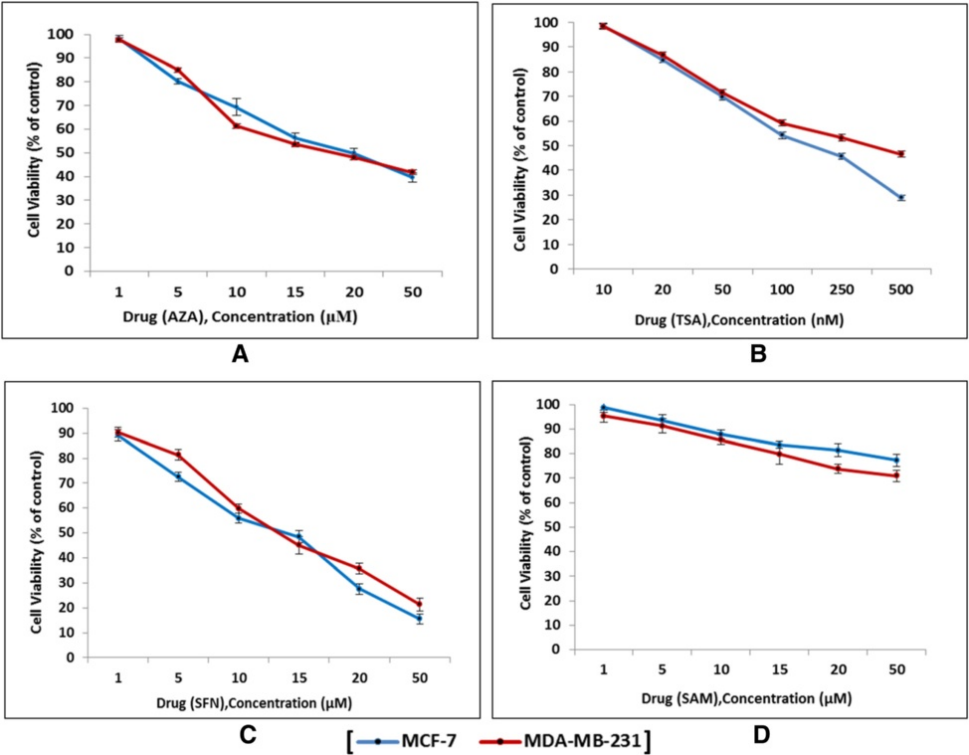
**MTT assay to determine the IC**_**50 **_**value of the different drugs and analyze their effect on cell viability.** The different drug concentrations used and the corresponding cell viability graphs are shown for AZA **(A)**, TSA **(B)**, SFN **(C)**, and SAM **(D)**. The IC_50_ value (that is, the concentration of drug which exhibited 50% cell viability for MCF-7 and MDA-MB-231 cells) were 15, 15, 10 μM, and 100 nM, respectively, for AZA, SAM, SFN, and TSA, respectively.

The effect of four different epigenetic modulators (that is, AZA, TSA, SFN, and SAM) on cell viability after 24 h treatment was assessed by colorimetric MTT assay in both the cell lines. The four modulators have their own distinct effect on cell viability at different concentrations. The cell survival level was observed to generally decrease with an increase in drug concentration indicating a dose-dependent behavior. An exception was seen in case of SAM treated cells where cell viability did not exhibit a significant decrease with regards to control untreated cells. The IC_50_ value (that is, the concentration of drug which results in 50% cell viability) were almost similar for both the cell lines - 15, 10 μM, and 100 nM for AZA, SFN, and TSA, respectively. A total of 15 μM of SAM was considered for treatment in both cell lines. Further experiments were performed with the above-mentioned drug concentrations (Figure 2).

### Expression profiles of DNMT and MBD genes after treatment with epigenetic modulators

**Figure 3:**
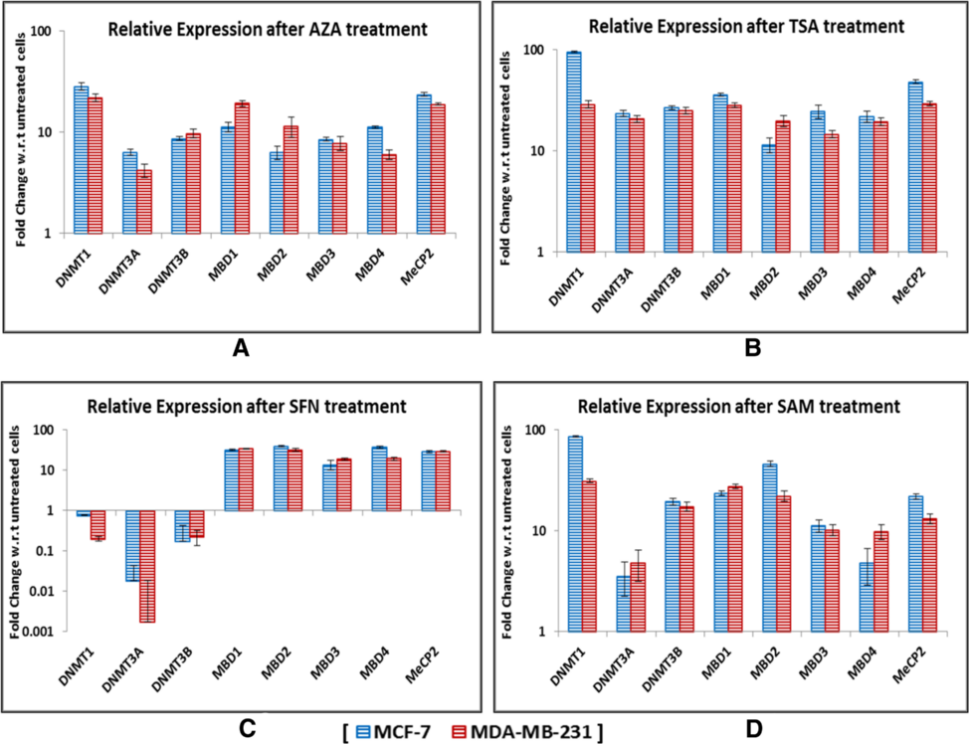
**Relative expression analysis of the various drug treated cells at the transcript level.** The effect of the epigenetic drugs - AZA **(A)**, TSA **(B)**, SFN **(C)**, and SAM **(D)** varied in different genes wherein the most significant increase in expression after AZA treatment was seen in DNMT1, after TSA treatment in MeCP2, after treatment with SAM in DNMT1 and in MBD1 after SFN treatment.

The effect of the epigenetic modulators on the expression of DNMT and MBD was determined by the quantitative analysis of mRNA for each of them in two different cell lines. In MCF-7, treatment with AZA resulted in an increase in expression of DNMT1 by 28.5-fold, of DNMT3A by 6.32-fold, of DNMT3B by 8.65-fold, and by 11.3-fold, 6.3-fold, 8.5-fold, 11.2-fold, and 23.6-fold for MBD1, MBD2, MBD3, MBD4, and MeCP2, respectively (Figure 3A). After treatment with TSA, the level of gene expression of DNMT1, DNMT3A, DNMT3B, MBD1, MBD2, MBD3, MBD4, and MECP2 increases by 95.1-fold, 23.5-fold, 26.7-fold, 36.2-fold, 11.4-fold, 24.6-fold, 21.95-fold, and 48.3-fold, respectively, with regards to untreated cells (Figure 3B). After treatment with SAM, the mRNA level of DNMT1, DNMT3A, DNMT3B, MBD1, MBD2, MBD3, MBD4, and MECP2 increases differently by 86-fold, 3.54-fold,19.4-fold, 23.6-fold, 46-fold,11.2-fold, 4.8-fold, and 21.8-fold, respectively (Figure 3D). In case of SFN treatment, there is an interesting observation. While the level of DNMTs shows drastic decrease, MBD genes are relatively highly expressed. There is downregulation of DNMT1 by 0.75-fold, of DNMT3A by 0.0185-fold, and DNMT3B by 1.174-fold. MBD1, MBD2, MBD3, MBD4, and MeCP2 show an increase of 31.2-fold, 39.5-fold, 13.6-fold, 36.8-fold, and 28.5-fold, respectively (Figure 3C).In case of MDA-MB-231 cells, after treatment with AZA, a known inhibitor of DNMTs, the transcript level of DNMT1 increases by 22.1-fold, of DNMT3A by 4.19-fold, and of DNMT3B by 9.69-fold, whereas there is an increase in expression by 19.3-fold, 11.5-fold, 7.8-fold, 5.98-fold, and 18.9-fold for MBD1, MBD2, MBD3, MBD4, and MeCP2, respectively (Figure 3A). The transcript level expression of DNMTs and MBDs also increases after treatment with TSA seen as 29-fold for DNMT1, 20.7-fold for DNMT3A, 25-fold for DNMT3B, 28.4-fold for MBD1, 19.8-fold for MBD2, 14.6-fold for MBD3, 13.8-fold for MBD4, and 29.5-fold for MeCP2 (Figure 3B). However after treatment with SFN, there is a difference between the expression levels of DNMTs and MBDs just as in case of MCF-7 cells. While the level of DNMTs is downregulated by 0.196-fold for DNMT1, 0.00172-fold for DNMT3A, and 0.224-fold for DNMT3B, the expression of MBDs increases by 33.9-fold for MBD1, 31.4-fold for MBD2, 18.7-fold for MBD3, 19.4-fold for MBD4, and 29.1-fold for MeCP2 (Figure 3C). Additionally after treatment with SAM, both the DNMT and MBD transcript levels show an increase by 31-fold for DNMT1, 4.79-fold for DNMT3A, 17.4-fold for DNMT3B, 27.5-fold for MBD1, 22-fold for MBD2, 10.2-fold for MBD3, 9.8-fold for MBD4, and 13.2-fold for MeCP2 (Figure 3D). The most significant increase in expression after AZA treatment is seen in DNMT1, in MeCP2 after TSA treatment, in DNMT1 after treatment with SAM, and in MBD1 after SFN treatment.

### Epigenetic modulators promote apoptotic cell death in breast cancer cells

**Figure 4:**
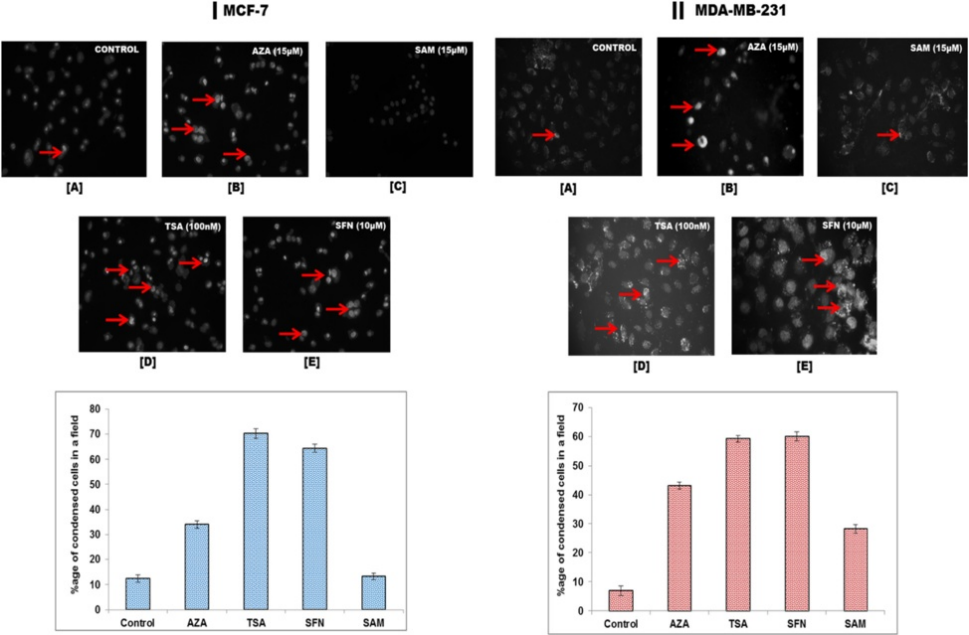
**MCF-7 and MDA-MB-231 cells were stained with Hoechst 33342 after treatment with AZA, TSA, SFN, and SAM for 24 h. [I]** The representative images of Hoechst 33342 stained nuclei and graphical representation of the percentage of condensed nuclei (n =3, mean ± S.D.) for MCF-7 cells. **[II]** The representative images of Hoechst 33342 stained nuclei and graphical representation of the percentage of condensed nuclei (n =3, mean ± S.D.) for MDA-MB-231 cells. *P* <0.05.

Chromatin condensation analysis by Hoechst staining was performed to study the cytotoxic effect of the epigenetic drugs on the MCF-7 and MDA-MB-231 cells (Figure 4I and II). During apoptosis, the chromatin becomes inert, highly condensed, undergoes fragmentation, and gets packaged into apoptotic bodies [34]. The morphological changes induced by apoptosis can be detected by the blue-fluorescent Hoechst 33342 dye which brightly stains the highly condensed, dense chromatin of apoptotic cells in comparison to the chromatin of non-apoptotic cells. After treatment with the epigenetic modulators at specific concentrations for 24 h - AZA (15 μM), TSA (100 nM), SFN (10 μM), and SAM (15 μM), the percentage of condensed nuclei are found to be 38.45%, 70%, 65.36%, and 11.30%, respectively, with control cells exhibiting 12.36% condensed nuclei in MC-7 cell line (Figure 4I). In case of MDA-MB-231 cells, 43.18%, 59.26%, 60%, and 28.30% condensed nuclei are observed after AZA, TSA, SFN, and SAM treatment, respectively, whereas the control cells exhibit 6.94% condensed nuclei (Figure 4II). Therefore, TSA and SFN are seen to be highly effective in inducing apoptosis in breast cancer cells.

### Higher protein expression corroborates the elevation in transcript level expression of DNMT in breast cancer cells

**Figure 5:**
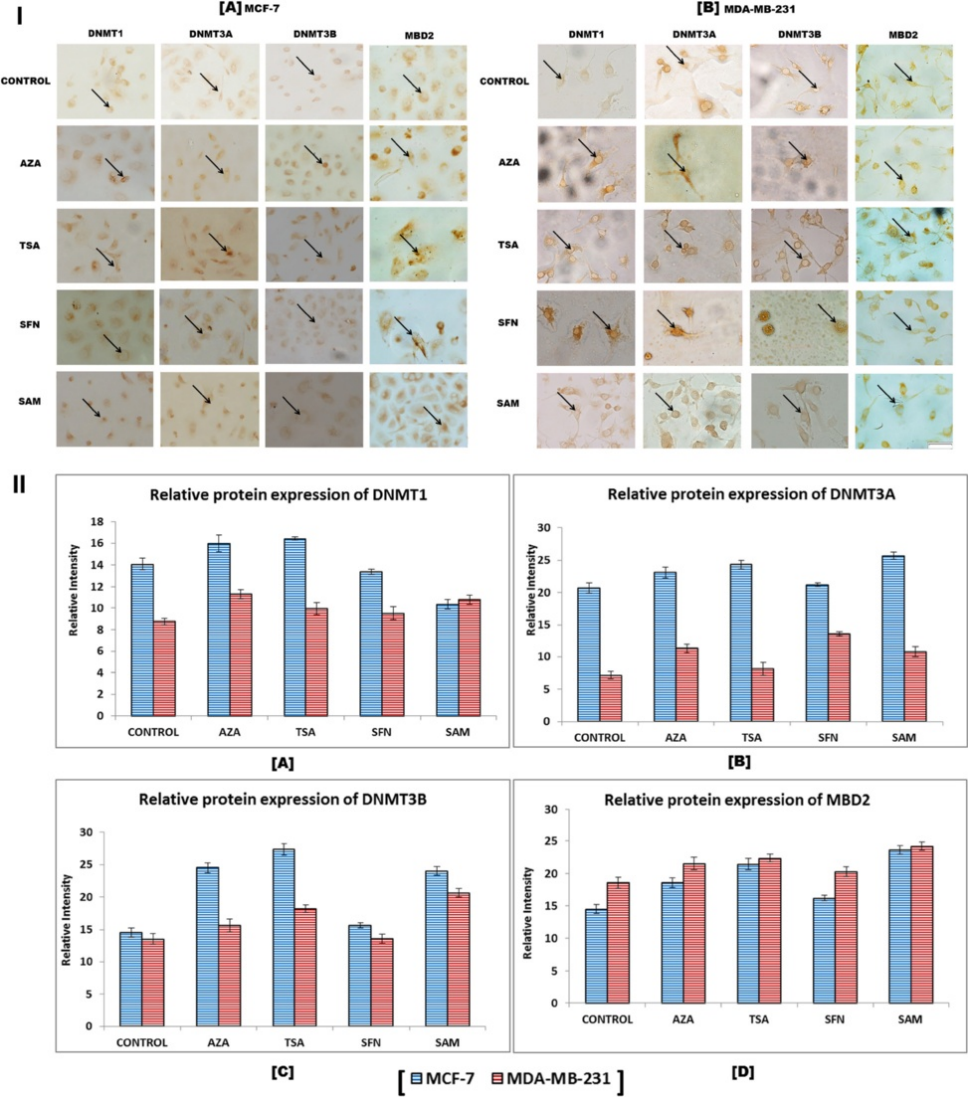
**Immunocytochemistry (40 X) of DNMT1, DNMT3A, DNMT3B, and MBD2. [I]** Representative images of antibody bound proteins show there is an increase in expression (arrow) after treatment with epigenetic modulators with respect to specific control (without treatment) cells in both the cell lines. **[II]** Graphical representation of the expression level different DNMT and MBD proteins by relative intensity using ImageJ software.

Immunocytochemical analysis was performed to study the effect of the epigenetic drugs and modulators on the level of expression of DNMT proteins. It is observed that in comparison to the untreated cells, the treated cells show higher expression of DNMT1, DNMT3A, DNMT3B, and MBD2 proteins which corroborates the elevated level of mRNA expression of DNMT and MBD proteins (Figure 5I and II). The other members of the MBD family also exhibit similar upregulation as MBD2 as shown in Figure 5.

### DNMT protein expression level is tissue stage-specific in breast cancer

**Figure 6:**
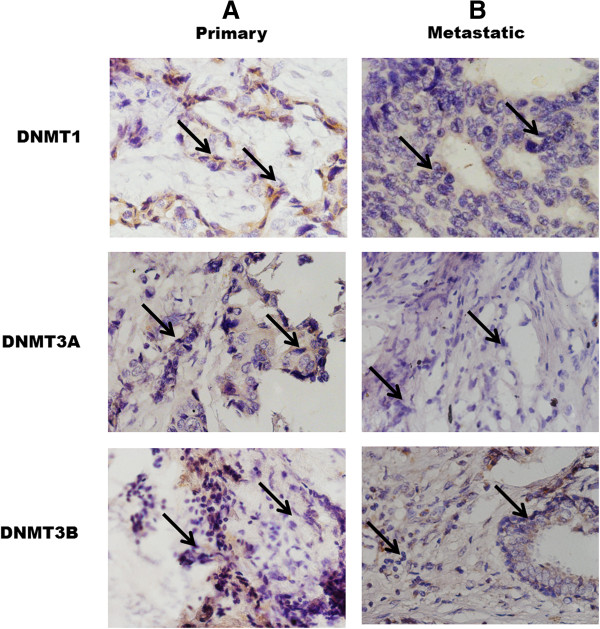
**Immunohistochemical analysis of the DNMT1, DNMT3A, and DNMT3B expression (20 X and 40 X magnifications). (A)** DNMT1, DNMT3A, and DNMT3B expression in primary breast cancer tissue samples. **(B)** DNMT1, DNMT3A, and DNMT3B expression in metastatic stage FFPE tissues shows higher protein expression (arrow) in comparison to primary stage. DNMT1 shows relatively higher expression than DNMT3A and DNMT3B in metastatic stage.

Similarly, the protein expression of DNMT proteins in FFPE breast cancer tissue samples was studied by immunohistochemistry. In all of the samples studied, there is significant expression of DNMT proteins. The level of DNMT1 is higher in the metastatic stage tissue sample in comparison to the primary stage (Figure 6A and B). In contrast, DNMT3A and DNMT3B show greater expression in the primary tissues with regards to metastatic stage (Figure 6A and B).

### Epigenetic modulators induce G_2_-M arrest and apoptosis in MCF-7 and MDA-MB-231 cells

**Figure 7:**
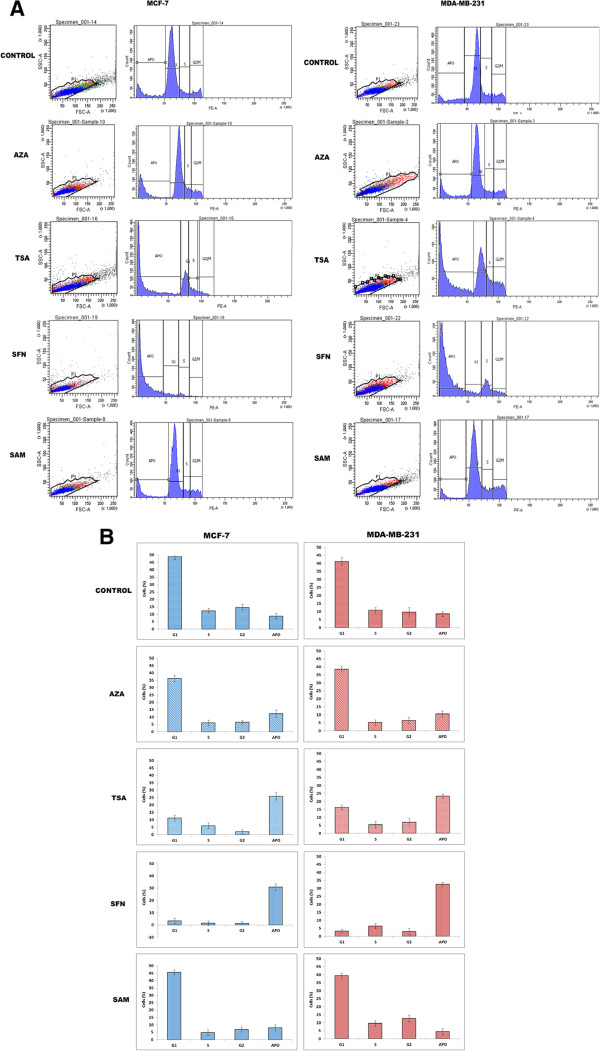
**FACS analysis of epigenetic modulator treated MCF-7 and MDA-MB-231 cells for 24 h to study the changes cell cycle distribution. (A)** AZA, TSA, SFN treatment shows G_2_/M phase arrest but TSA, SFN treatment were much more effective than AZA. **(B)** Graphical representation of G_1_, S, G_2_/M, and APO population percentage in breast cancer cells (n =3, mean ± S.D.). *P* <0.05.

In order to study the effect of the epigenetic drugs and modulators on the cell cycle and cell growth, flow cytometry based cell cycle analysis after treatment with the epigenetic modulators was performed (Figure 7A). There is an alteration in the cell cycle distributions in both MCF-7 and MDA-MB-231 cells after treatment with various epigenetic factors for 24 h. When MCF-7 control (untreated) cells were analyzed, percentage of G_1_, S, G_2_/M, and apoptotic cells were found to be 58.5%, 12.3%, 14.6%, and 8.8%, respectively (Figure 7A(a)). After treatment with AZA (15 μM), the percentage of G_1_, S, G_2_/M, and apoptotic cells is found to be 36.3%, 6.0%, 6.5%, and 12.3%, respectively (Figure 7B(a)). When cells were treated with TSA, percentage of G_1_, S, G_2_/M, and apoptotic cells changed drastically to be 11.2%, 5.4%, 2.0%, and 25.9%, respectively, with regards to untreated cells (Figure 7B(a)). Similar changes are also observed after SFN treatment with 4.2% cells in G_1_, 1.4% cells in S, 1.2% cells in G_2_, and 30.9% cells in apoptotic phase. However, SAM treatment resulted in 45.7%, 4.7%, 6.9%, and 8.0% cells in G_1_, S, G_2_/M, and apoptotic population, respectively.

In case of untreated MDA-MB-231 control cells, percentage of G_1_, S, G_2_/M, and apoptotic cells is 41.2%, 10.7%, 9.6%, and 8.5%, respectively (Figure 7B(b)). After treatment with AZA (15 μM), the percentage of G_1_, S, G_2_/M, and apoptotic cells is found to be 41.1%, 5.1%, 6.4%, and 10.5%, respectively (Figure 7B(b)). Similarly, for TSA (100 nM) treatment, the percentage of G_1_, S, G_2_/M, and apoptotic cells is observed to be 16.3%, 5.4%, 6.9%, and 23.3%, respectively, with regards to untreated cells (Figure 7B(b)). The percentage of G_1_, S, G_2_/M, and apoptotic cells after treatment with SFN is seen to be 3.2%, 6.3%, 2.9%, and 32.7%, respectively with regards to untreated cells (Figure 7B(b)). Also, after SAM treatment, 47.6% of cells in G_1_ phase, 9.6% of cells in S phase, 12.7% of cells in G_2_/M, and 4.3% of apoptotic cells are reported with regards to untreated cells (Figure 7B(b)). From the above results, it is clear that SFN is most effective in inducing apoptosis in both MCF-7 and MDA-MB-231 cells as is evident from the highest percentage of apoptotic cells in case of SFN treated cells with regards to untreated cells.

## Discussion

The epigenetic gene-silencing machinery in the cell executes its function via efficient co-ordination between the two main protagonists - DNMT and MBD proteins. While DNA methylation ensures that the genes are effectively tagged for inactivation, MBD proteins recognize these ‘OFF’ marks and result in chromatin compaction with the help of histone modifying enzymes and chromatin remodeling complexes. In recent years, epigenetic causes are being considered as important hallmarks for malignant transformation. In this context, targeted inhibition of DNMT and MBD proteins is now considered as a means of unmasking silenced genes to counterattack malignant transformation.In the present study, we have analyzed the gene and protein expression profile of DNMT and MBD proteins in MCF-7 and MDA-MB-231 breast cancer cell lines after treatment with known epigenetic modulators such as DNMT inhibitor - AZA, Methyl donor - SAM, and HDAC inhibitors - TSA and SFN. We have also investigated the effect of these drugs on cell viability, cell cycle, and cell growth in breast cancer cells. The epigenetic modulators affect the cell growth of both the cell lines in a dose-dependent manner, that is, with increasing concentrations of drugs; the cell viability gradually decreases (Figure 2A, B, C, and D). It can thus be assumed that epigenetic modulators may affect and modify the epigenetic modifications in various cell cycle regulatory genes which could impact the cell viability of breast cancer cells.

We have observed that after treatment with epigenetic drugs, the level of transcript expression of MBDs has consistently increased for every drug treatment in both the cell lines (Figure 3A, B, C, and D); however DNMTs exhibit decrease in expression in comparison to untreated cells after treatment with SFN (Figure 3C). The decrease in DNMT expression after SFN treatment is consistent with the results obtained by Hsu *et al*., in 2011, where they have demonstrated that SFN significantly decreased DNMT1 and 3A mRNA expression and that SFN also impacts global DNA methylation and site-specific demethylation of the cyclin D2 promoter [35]. Keeping the above findings in mind, it can be suggested that SFN has the ability to affect DNA methylation either independently via a yet unknown molecular mechanism or via association with histone deacetylases by interrupting the MBD mediated gene-silencing circuit.

The increase in level of gene expression is substantiated at the protein level. The results of immunocytochemistry demonstrate that epigenetic modulator treated cells show higher expression of DNMT1, DNMT3A, DNMT3B, and MBD proteins in comparison to untreated cells in both the cell lines (Figure 5I and II). In case of tissue samples, the expression of different DNMTs is tissue stage-specific. DNMT1 exhibits significantly higher expression in the metastatic stage, whereas DNMT3A and DNMT3B have higher expression in the primary stage in comparison to the metastatic samples (Figure 6A and B). This observation may be indicative of the fact that DNMTs have differential role to play during initiation, maintenance and progression of neoplasia. It is already reported that DNMT3A and DNMT3B are *de novo* methyltransferases which mainly add methyl groups to the cytosine bases of the newly synthesized hemimethylated daughter strands at the replication foci [5,6]. Additionally, DNA hypermethylation-induced gene silencing is a triggering event during tumorigenic transformation [21,36,37]; hence DNMT3A and DNMT3B are essentially required at this stage to methylate promoter CpG islands adjacent to transcription start sites of tumor-related genes, cell-cycle regulatory, and DNA repair genes. Therefore, increased expression of DNMT3A and DNMT3B in the primary stages rather than the metastatic stage (Figure 6A and B) validates this information. Although, many of the key gene-silencing events occur very early during the premalignant stages of tumor progression, the process of epigenetic gene silencing continues through the entire progression of human cancer, where DNMT1 plays the predominant role as the maintenance methyltransferase. Hence, the elevated level of DNMT1 in the metastatic stage (Figure 6A and B) is a confirmation of the above finding.

MBD proteins are known to interact with methylated DNA in concert with HDACs to repress transcriptional activity via heterochromatin formation. As the HDAC inhibitors effectively trap HDAC and prevent them to associate with MBD proteins, there is a possibility that the action of MBD proteins can be disrupted. If the activity of MBD proteins is disrupted, then DNMT mediated hypermethylation and gene silencing can also be effectively hindered. Based on this assumption, MCF-7 and MDA-MB-231 cells were treated with IC_50_ concentration of the epigenetic drugs - AZA (15 μM), TSA (100 nM), SFN (10 μM), and SAM (15 μM) to study their effect on cell cycle and cell growth. It is observed that all the epigenetic modulators promote apoptotic cell death as is evident form increased chromatin condensation which is a distinct characteristic of apoptotic cells (Figure 4I and II). The percentage of condensed nuclei is highest in TSA and SFN treated cells (Figure 4I and II), thus these two modulators are more effective in inducing apoptotic cell death. On further analysis of the effect of these modulators on cell cycle, it is seen that in comparison to control untreated cells, cells treated with AZA and SAM, show increase in G_1_-phase cells, decreased percentage of S and G_2_ population as well as increase in apoptotic cells (Figure 7A and B). Additionally, cells treated with TSA and SFN exhibit reduction in G_1_ phase cells, decrease in percentage of G_2_ population and drastic increase in apoptotic cell population (Figure 7A and B). Thus, TSA and SFN affect all the stages of cell cycle, arresting cell progression in each successive stage and ultimately increasing the rate of apoptosis in the cell population. From the above results, it is clear that the epigenetic modulators - AZA, TSA, SFN, and SAM induce differentiation, growth arrest, and apoptosis in breast cancer cells.

The current work has significantly established that epigenetic modulators such as DNMT and HDAC inhibitors can indirectly affect the methylation mediated gene-silencing machinery by directly targeting the DNMTs and HDACs and thus affecting the expression of DNMTs and MBDs. The findings of the study indicate that the epigenetic modulators AZA, TSA, SFN, and SAM affect the cell growth, viability, and apoptosis rate in breast cancer cells. Previously, Mirza *et al.* had demonstrated how natural polyphenols can modulate the expression of DNMT proteins in breast cancer patients [38]. In lieu of their analysis, we targeted both DNMTs and MBDs, the crucial elements in the gene-silencing machinery via epigenetic modulators. The current study for the first time showcases a direct approach for targeting the gene-silencing machinery during malignant transformation. However, in-depth mechanistic studies should be carried out to elucidate how these compounds affect the gene transcription as the above drugs act at the protein level. The study reinforces the view that these epigenetic agents can induce cancer inhibitory activity and may be useful in investigating epigenetic sources of cancer treatment.

## Conclusions

As translational research in the field of cancer epigenomics is increasingly focusing its attention towards achieving epigenetic therapies for cancer treatment, targeting the DNA methylation-based gene-silencing machinery assumes great significance. In this study, we have revealed the effect of various epigenetic modulators on the gene and protein level expression of DNMTs and MBD proteins. We have also demonstrated how these modulators inhibit cell growth, affect cell cycle by blocking G_2_-M progress and induce apoptotic cell death. This study has demonstrated the significance of targeting DNA methylation, histone modification, especially, histone deacetylation and MBD proteins, all of which concomitantly act to induce gene silencing and transcriptional incompetence. The targeted inhibition of MBD function via DNMT and HDAC inhibitors is thus a promising therapeutic option for efficient treatment of neoplastic progression.

## Methods

### *In vitro* cell culture and drug treatment

Human breast carcinoma cell lines MCF-7 and MDA-MB-231 were obtained from ATCC through NCCS, Pune, India. The cells were cultured and maintained in Modified Eagle’s Medium (MEM) and Dulbecco’s Modified Eagle’s Medium (DMEM), respectively, supplemented with 10% (v/v) Fetal Bovine Serum (FBS) and 100 IU/mL Penicillin and 0.1 mg/mL streptomycin in a humified atmosphere of 5% CO_2_ at 37°C. Stock solutions of AZA (Sigma), TSA (Sigma), and SFN (Sigma) were prepared in dimethylsulphoxide (DMSO) whereas SAM (Sigma) was dissolved in milli-Q water. Stock solutions were further diluted to working concentrations in DMEM prior to use. Cells were harvested by trypsinization and cell number was counted by hemocytometer. The number of living cells was calculated by Trypan blue staining (0.2% v/v). For determining the concentration of drug that inhibited cell proliferation by 50% (IC_50_), 5 X 10^3^ cells per well were seeded in a 96-well microtiter plates and after 24 h incubation, were treated with the epigenetic modulators at different concentrations (AZA, SAM, SFN (1, 5, 10, 15, 20, 50 μM) and TSA (10, 20, 50, 100, 250, 500 nM)) mixed in respective medias supplemented with 5% FBS. Control cells were treated with DMSO only.

### Cell viability analysis by colometric MTT assay

The effect of the epigenetic modulators, DNMT and HDAC inhibitors on cellular proliferation was assessed by 3-(4, 5-Dimethylthiazol-2-yl)-2, 5-Diphenyltetrazolium Bromide (MTT) assay, using standard protocol. Briefly, the drug-treated cells in each of the 96 wells were washed twice with PBS. 0.8 mg/mL MTT solution was prepared from stock MTT solution (5 mg/mL PBS, pH 7.2). A total of 100 μL MTT solution was added to each well and incubated at 37°C for 4 h in dark. The supernatant was removed and 100 μL of DMSO was added into each well to dissolve the formazan crystals. The absorbance was measured at 570 nm and results were expressed as the mean of three replicates as a percentage of control (taken as 100%). The extent of cytotoxicity was defined as the relative reduction of the optical density (OD), which correlated to the amount of viable cells in relation to cell control (100%). The cell viability was plotted in a graph and the IC_50_ was calculated accordingly to decide the optimum dosage of the drugs for further studies.

### Relative gene expression analysis after drug treatment by real-time PCR

MCF-7 and MDA-MB-231 cell lines were treated with sub lethal dosages of AZA (15 μM), TSA (100 nM), SFN (10 μM), and SAM (15 μM) for 24 h. After treatment for the required time, total cellular RNA was extracted with TriReagent (Sigma) according to the manufacturer’s instructions. qRT-PCR was performed using cDNA prepared from 1 μg of total RNA prepared using RevertAid First Strand cDNA Synthesis Kit (Thermo Scientific) and SYBR® Green JumpStart™ Taq ReadyMix in the Realplex^4^Eppendorf system. The mRNA level was normalized to β-actin, as described earlier [39]. The primer sequences are provided in Table 1.

**Table 1:** List of sequence and product length of the real-time PCR primers used in this study

**Gene**	**Primer sequence**		**Product length (bp)**
DNMT1	F	5′-GGCTGAGATGAGGCAAAAAG -3′	112
	R	5′-ACCAACTCGGTACAGGATGC -3′	
DNMT3A	F	5′-TATTGATGAGCGCACAAGAGAGC -3′	111
	R	5′-GGGTGTTCCAGGGTAACATTGAG -3′	
DNMT3B	F	5′-AATGTGAATCCAGCCAGGAAAGGC -3′	191
	R	5′-ACTGGATTACACTCCAGGAACCGT -3′	
MeCP2	F	5′-TGACCGGGGACCCATGTAT -3′	145
	R	5′- CTCCACTTTAGAGCGAAAGGC -3′	
MBD1	F	5′- CCTGGGTGCTGTGAGAACTGT -3′	107
	R	5′- TTGAAGGCAATTCTCTGTGCTC -3′	
MBD2	F	5′- AGGTAGCAATGATGAGACCCTTTTA -3′	116
	R	5′- TAAGCCAAACAGCAGGGTTCTT -3′	
MBD3	F	5′- CCGCTCTCCTTCAGTAAATGTAAC -3′	101
	R	5′- GGCTGGAGTTTGGTTTTCAGAA -3′	
MBD4	F	5′- AGACCCGCCGAATGACCT -3′	144
	R	5′- GCACCAAACTGAGCAGAAGCG -3′	
β-ACTIN	F	5′- CTGGAACGGTGAAGGTGACA -3′	140
	R	5′- AAGGGACTTCCTGTAACAACGCA -3′	

### Chromatin condensation analysis by Hoechst staining

After treatment with epigenetic modulators, cells were stained with Hoechst 33342 stain (1 mg/mL, Invitrogen) followed by incubation for 10 min at 37°C. Images were taken under UV filter using Epi-fluorescent Microscope (Olympus IX71) at 400 X magnification with an excitation wavelength of 355 to 366 nm and an emission wavelength of 465 to 480 nm. Condensed nuclei were counted against total number of nuclei in the field, and the percentage of apoptotic nuclei were calculated and plotted graphically.

### Immunocytochemistry

Immunocytochemistry was performed as per our previous protocol with some modifications [39-43]. In brief, MCF-7 and MDA-MB-231 cells were grown on glass coverslips and treated with AZA (15 μM), SAM (15 μM), TSA (100 nM), and SFN (10 μM) for 24 h. The treated cells were fixed by ice cold methanol and permeabilized by 0.25% triton X-100 in PBS. Cells were incubated with 1% BSA in PBST for 30 min to block non-specific binding of the antibodies. The endogenous peroxidase activity was blocked by incubating in 5% H_2_O_2_ in methanol for 20 min followed by incubation in primary antibodies for DNMT1, DNMT3A, and DNMT3B overnight at 4°C. The cells were then washed in PBS and incubated with HRP-conjugated anti-rabbit secondary antibody (Santa Cruz Biotech) for 1 h followed by another wash. Finally, reactions were visualized by incubation with 3, 3′-Diaminobenzidine (DAB, substrate and chromogen, Sigma) and counterstained with Mayer’s hematoxylin. For negative control, cells were incubated overnight with dilution buffer (no primary antibody) [37].

### Immunohistochemistry

Twenty formalin-fixed paraffin-embedded (FFPE) breast tissue samples were collected from Drs. Tribedi & Roy Diagnostic Laboratory (Kolkata, India). Of these samples, two were cancer adjacent tissues whereas the rest of them were cancerous tissues. Of the eighteen tumor tissues, ten were primary stage and eight were metastatic stage breast tissues. FFPE blocks were sliced into 0.5 μM thin slices and subjected to antigen retrieval with tris-EDTA buffer, endogenous peroxidase blocking, and rinsed with tris-buffered saline (TBS) containing 0.025% Triton X-100 (TBS-T). Rabbit polyclonal anti-DNMT1 (Santa Cruz), rabbit polyclonal anti-DNMT3A (Santa Cruz), rabbit polyclonal anti-DNMT3B (Santa Cruz), and rabbit polyclonal anti-DNMT3B (Santa Cruz) were used as primary antibodies. The secondary antibody used was anti-rabbit (Invitrogen). After incubation with primary antibodies at 4°C overnight, the specimens were rinsed with TBS and incubated at room temperature for 1 h with secondary antibody. After rinsing with TBS, all specimens were color-developed with DAB.

### Cell cycle analysis by FACS

Flow cytometry analysis of Propidium Iodide (PI) stained nuclei was done to assess the effect of epigenetic drugs on the cell cycle distribution. AZA (15 μM), SAM (15 μM), TSA (100 nM), and SFN (10 μM) treated cells were incubated in respective media with 5% FBS for 24 h. The cells were then trypsinized, collected by centrifugation (500 × g for 5 mins at 4°C), washed twice with PBS and then fixed in 90% ice-cold methanol. After incubation at -20°C for 1 h, cells were centrifuged and resuspended in PBS followed by treatment with RNaseA (500 U/mL) to digest the residual RNAs and stained with PI (10 μg/mL). Samples were incubated for 30 min at 4°C and cell cycle analysis was performed with a Becton-Dickinson fluorescence-activated cell sorter (FACS).

### Statistical analysis

All data are presented as means ± SD. Statistical analysis was performed using the Student’s t-test by SPSS software. Values of *P* <0.05 were considered as significant value.

## Abbreviations

AZA: 5-Aza-2′-deoxycytidine; DMEM: Dulbecco’s Modified Eagle’s Medium; DNMTs: DNA Methyltransferases; DMSO: Dimethylsulphoxide; FBS: Fetal Bovine Serum; FACS: Fluorescence-Activated Cell Sorter; FFPE: Formalin-Fixed Paraffin-Embedded; HDAC: Histone Deacetylase; MBD: Methyl-CpG-Binding Domain; MEM: Modified Eagle’s Medium; MTT: 3-(4 5-Dimethylthiazol-2-yl)-2, 5-Diphenyltetrazolium Bromide; PI: Propidium Iodide; SAH: S-Adenosyl Homocysteine; SAM: S-Adenosyl Methionine; SFN: Sulforaphane; TBS: Tris-Buffered Saline; TSA: Trichostatin A.

## Competing interests

The authors declare that they have no competing interests.

## Authors’ contributions

SKP, SK, and DS conceived the project and wrote the manuscript; SK and DS performed the experimental works on MTT assays, RT-PCR, FACS, and chromatin condensation in the two different cell lines; MD carried out the immunocytochemistry and immunohistochemistry; AS and SP participated in the data representation; SR and NP performed the statistical analysis. MD, AS, SP, SKR, NP, and MR helped in collecting information and literature survey, in preparing the figures and tables; all the authors critically commented on the manuscript and took part in discussion. SKP edited the final version of the manuscript. All authors read and approved the final manuscript.
